# Three-year results from the Retro-IDEAL study: Real-world data from diabetic macular edema (DME) patients treated with ILUVIEN^®^ (0.19 mg fluocinolone acetonide implant)

**DOI:** 10.1177/1120672119834474

**Published:** 2019-03-18

**Authors:** Albert J Augustin, Silvia Bopp, Martin Fechner, Frank Holz, Dirk Sandner, Andrea-M Winkgen, Ramin Khoramnia, Thomas Neuhann, Marcus Warscher, Martin Spitzer, Walter Sekundo, Berthold Seitz, Tobias Duncker, Christian Ksinsik, Helmuth Höh, Daniela Gentsch

**Affiliations:** 1 Augenklinik, Department of Ophthalmology, Staedtisches Klinikum Karlsruhe, Karlsruhe, Germany; 2 Augenklinik Universitätsallee, Bremen, Germany; 3 Augenklinik Stralsund, Stralsund, Germany; 4 Universitäts-Augenklinik Bonn, Bonn, Germany; 5 Universitäts-Augenklinik Dresden, Germany; 6 Augenzentrum Lüdenscheid, Germany; 7 Universitäts-Augenklinik Heidelberg, Heidelberg, Germany; 8 MVZ Augenzentrum, München, Germany; 9 Klinik für Augenheilkunde, Klinikum Frankfurt Höchst, Frankfurt, Germany; 10 Universitäts-Augenklinik Hamburg Eppendorf, Hamburg, Germany; 11 Universitäts-Augenklinik, Marburg, Germany; 12 Universitäts-Augenklinik des Saarlandes, Homburg/Saar, Germany; 13 Makula Zentrum, Institut für Augenheilkunde Halle, Halle, Germany; 14 Augenklinik am Glacis, Torgau, Germany; 15 Augenklinik, Dietrich-Bonhoeffer-Klinikum, Neubrandenburg, Germany; 16 Augenpraxisklinik Konstanz, Konstanz, Germany

**Keywords:** Steroid, ILUVIEN, DME, microdosing, Retro-IDEAL, 3 years

## Abstract

**Introduction:**

The Retro-IDEAL (ILUVIEN Implant for chronic DiabEtic MAcuLar edema) study is a retrospective study designed to assess real-world outcomes achieved with the ILUVIEN® (0.19 mg fluocinolone acetonide (FAc)) in patients with chronic diabetic macular edema (DME) in clinical practices in Germany.

**Methods:**

This study was conducted across 16 sites in Germany and involved 81 eyes (63 patients) with persistent or recurrent DME and a prior suboptimal response to a first-line intravitreal therapy (primarily anti-VEGF intravitreal therapies).

**Results:**

Patients were followed-up for 30.8 ± 11.3 months (mean ± standard deviation) and had a mean age of 68.0 ± 10.4 years. Best-recorded visual acuity (BRVA) improved by +5.5 letters at month 9 (P ⩽ 0.005, n=56; from a baseline of 49 letters) and this was maintained through to month 30 (P ⩽ 0.05, n = 42). There was a concurrent improvement in central macular thickness with a reduction from 502 µm at baseline to 338 µm at year 1 (P ⩽ 0.0001, n = 43). This effect was sustained to year 3 (i.e. 318 µm; P ⩽ 0.0001, n = 29). Mean intraocular pressure (IOP) remained constant between baseline and year 3 with a peak change of 1.9 mm Hg occurring at year 1. Elevated IOP was observed in a similar percentage of patients prior to (22.2% of cases) and following (27.2%) treatment with the FAc implant. In the majority of cases, these elevations were managed effectively with IOP medications.

**Conclusions:**

Despite substantial amounts of prior intravitreal treatments – primarily with anti–vascular endothelial growth factor (VEGF) drugs – this real-world study showed that sustained structural and functional improvements can last for up to 3 years with a single FAc implant.

## Introduction

Diabetic retinopathy (DR) is an important microvascular complication and is the leading cause of blindness in the working-age population of developed countries.^1–3^ The biochemical pathways underpinning the pathogenesis of DR lead to the breakdown of the blood retinal barrier and the development of diabetic macular edema (DME), which is largely driven by the consequential effects of increased oxidative stress, inflammation and vascular dysfunction, all of which have been described in human and animal models of DR.^4^ In terms of functional effects, there is increasing evidence to suggest that neuronal changes^5^ may explain, in part, the early defects observed in DR, which include loss of colour vision, loss of contrast sensitivity, abnormalities in the electroretinogram and visual field defects. Neuronal dysfunction in DR is probably due to many biochemical changes, including impaired glutamate metabolism, loss of synapses and dendrites and apoptosis of ganglion cells. Another early event is increased retinal leukostasis, which is significantly increased in an animal model of DR,^6^ and, instead of an acute vasculitis, inflammation in this case has been described as a sustained, chronic inflammation. This means that anti–vascular endothelial growth factor (VEGF) therapy can be highly effective in the early phase but, as DME progresses towards the later phase, anti-inflammatory agents such as corticosteroids become more effective and anti-VEGF therapy becomes less effective. Clinicians therefore need to tailor treatment towards corticosteroid therapy once patients are not responding sufficiently to anti-VEGF therapy (~40% had <5 letter change at 3 months in the EARLY analysis).^7^

The decline in responsiveness to anti-VEGF therapy has been reported in the pivotal studies for ranibizumab.^8^ In the RISE and RIDE studies, trials of ranibizumab injection in patients with clinical significant macular edema secondary to diabetes mellitus, patients who received monthly 0.5 mg ranibizumab injections showed a mean improvement of 11.1 Early Treatment Diabetic Retinopathy Study (ETDRS) letters at 12 months, and this was maintained up to 36 months. In the sham arm, however, patients only qualified for monthly 0.5 mg ranibizumab (i.e. switched from sham to treatment) at month 24. This group gained only 2.8 ETDRS letters between 24 and 36 months.^8^ Thus, it can be concluded that the development of persistent or recurrent retinal edema occurring in the sham arm may result in a certain amount of potential vision gain being lost irreversibly.

Clearly, once DME has progressed to the point where anti-VEGF therapy is no longer offering adequate efficacy, a different therapeutic approach is required. In contrast to anti-VEGF therapy, which is focused on reducing levels of VEGF only, corticosteroids have a broader range of action. In addition to some anti-VEGF efficacy, they can also lower aqueous humour levels of several pro-angiogenic and inflammatory cytokines including interleukin-6 (IL-6), interferon-induced protein-10 (IP-10), monocyte chemotactic protein-1 (MCP-1) and platelet-derived growth factor-AA (PDGF-AA).^9^ This broad range of anti-inflammatory actions results in corticosteroids being better suited than anti-VEGF therapy to counteract the inflammatory phase of DME.

The long-acting fluocinolone acetonide implant (FAc implant; ILUVIEN^®^, Alimera Sciences, Inc., Alpharetta, GA, USA)^10,11^ is approved in many countries for the treatment of visual impairment associated in patients with persistent or recurrent DME. The implant is injected into the vitreous where it releases a microdose of FAc (0.2 µg per day) continuously for up to 3 years with the aim of reducing macular edema and improving vision.

Importantly, the continuous dosing also assures that treatment is maintained steadily even in the event of delays to follow-up appointments. This is an important benefit as many factors – including suboptimal patient compliance and clinical capacity issues – can disrupt the intensive dosing schedule required for optimal results in anti-VEGF therapy.^12,13^

The FAc implant has an anticipated lifespan of up to 3 years and has now been available in Germany for longer than this. As a result, it is possible for the first time to evaluate the safety and efficacy of the implant in everyday clinical practice throughout its anticipated lifespan. Moreover, at the time of the Fluocinolone Acetonide for Diabetic Macular Edema (FAME) trials, grid laser was the main DME therapy, whereas the current standard of care is predominantly intravitreal injections of anti-VEGF agents, and thus it is possible to assess the safety and effectiveness of the implant after prior intravitreal therapies. For these reasons, this retrospective evaluation of real-world outcomes with the FAc implant during a 3-year period of treatment in Germany has been performed and results from this Retro-IDEAL (ILUVIEN Implant for chronic DiabEtic MAcuLar edema) study are reported here.

## Methods

### Subjects and methods

This study was conducted in accordance with the Declaration of Helsinki and Germany’s Data Protection Act (AMG §4, (23), Satz 3). Following written approval from each of the 16 centres and written consent being obtained from each patient, anonymised data were extracted. Data were then retrospectively collected from the 16 clinical sites by ANFOMED GmbH (Moehrendorf, Germany). Digital templates were used to record and document the findings at each clinical site and were compiled to enable analyses to be performed on the overall group.

The present analysis was based on data extracted between September 2016 and September 2017. Data from adult patients (minimum 18 years) who had received the 0.2 μg/day FAc implant for the treatment of DME were identified (although in some cases off-licence indications were entered) at each participating centre and extracted from each site, before being pseudoanonymised prior to amalgamation into a single dataset. The datasets were searched to identify any patient treated with 0.2 μg/day FAc implant. Data were then extracted from the entire record of eligible patients so that observations and treatments before and after 0.2 μg/day FAc treatment were included. Prior to ILUVIEN, a 12-month period was defined (i.e. a pre-ILUVIEN treatment phase) so that DME therapies in this period could be standardised by time. Treatment was carried out according to the clinicians’ discretion, and this is denoted by the range of visual acuities defined in the DME population (20–80 ETDRS letters). The decision to switch to the 0.2 μg/day FAc implant was recorded and based on at least one of the following: (1) insufficient efficacy (i.e. suboptimal) during the pre-treatment phase; (2) strong edema (i.e. persistent); (3) no reaction (i.e. response) of visual acuity (VA) to the pre-treatment and (4) high number (i.e. ⩾1 recurrence to first-line therapies) of recurrences during the pre-treatment phase.

### Data extracted and analysis

Data were extracted from patients with a minimum follow-up of 30 months and the following parameters were analysed:

1. Baseline demographics, disease characteristics (including concomitant diseases) and DME treatments. This included patient data (age, sex, body mass index (BMI)); history of disease and history of DME treatments (i.e. diagnosis and date of diagnosis, the eye treated, the type of therapy (laser, intravitreal injections, vitrectomies), lens status, DR score (measuring using fluorescence angiography))
2. Prior treatments for intraocular pressure (IOP) and DME
3. Injection of the 0.2 μg/day FAc implant (the date, the eye treated, the indication prescribed)
4. VA outcomes (converted from Snellen fraction to an ETDRS letters score, best recorded VA)^14^
5. Central macular thickness (CMT; measured using Topcon 3D OCT-2000, Heidelberg Spectralis, Carl Zeiss Cirrus, Optovue RTVue 100)
6. Supplemental ocular treatments administered after the 0.2 μg/day FAc implant was injected
7. The incidence of IOP elevation (IOP change of >10 mm Hg from baseline and an increase above 30 mm Hg) and management (use of IOP-lowering medication to control IOP)
8. Adverse events including cataract formation and surgical removal times

### Data analysis

Baseline values for VA, CMT and IOP are the last non-missing values taken on or before the initial date that the 0.2 μg/day FAc implant was injected. Every non-missing assessment collected after its administration was assigned a follow-up visit number as follows: visit 0 (baseline or day 0), 1 (day 1 to 90), 2 (day 91 to 180) and so on up to visit 12 (36 months). If more than one assessment was documented within one visit period, the mean of the values was calculated at time zero and then every 3 months. If no follow-up was recorded in one visit, it was excluded from the analysis. Drug usage was recorded in the 12 months prior to the FAc implant (at 12 months and 6 months) and during the 36 months after (visits 0 to 12) the 0.2 μg/day FAc implant was administered.

### Statistical analysis

Data were included in the analyses only if both baseline and follow-up data were available for each patient. Data were reported as mean ± standard deviation (SD) unless otherwise stated. Statistical testing was performed using Wilcoxon signed-rank test and significance was taken as P < 0.05 and values at reported time points were compared against baseline levels.

### Exclusion

A two-step approach was used to identify DME patients – patients with no diabetes diagnosed and diabetic patients with no diagnosis for DME. Finally, data from these patients were excluded if only baseline data were available.

## Results

### Demographics (all patients in Retro-IDEAL study)

Data from a total of 94 eyes (from 76 patients) were extracted from the clinics’ records. The patients had a mean age of 66.9 ± 12.2 years (48.2% were aged >70 years) and the majority (74%) of eyes were pseudophakic. The mean follow-up period was 30.8 ± 11.3 months. Diagnoses mainly included DME (n = 83; time since diagnosis, 3.7 ± 2.9 years), although some off-label usage was reported in cystoid macular edema (n = 6; 4.0 ± 4.0 years), retinal vein occlusion (n = 1; 2.2 years) and uveitis (n = 5; 4.6 ± 5.5 years).

### Demographics (all DME patients in Retro-IDEAL study)

Among the 94 eyes for which data were extracted, 81 eyes diagnosed with DME had been analysed based on the above inclusion criteria. The demographic details for these 81 eyes (from 63 patients) are summarised in Table 1. The mean age of the patients was 68.0 ± 10.4 years and the majority of eyes were pseudophakic (75.3%) and had a concomitant disease (76.5%). The eyes had been diagnosed with DME for 3.8 ± 2.9 years and they had been heavily pre-treated in the 12 months preceding FAc implantation – 92.5% had received laser therapy, 97.5% had received intravitreal therapy (predominantly anti-VEGF – ranibizumab (91.1%) and bevacizumab (44.3%) – or intravitreal triamcinolone acetonide (41.7%)) and 48% had undergone a vitrectomy (Table 1). In 39.2% of cases, a combination of all three treatments (laser, anti-VEGF and steroid) had been administered prior to the FAc implant and in 43.2% of cases a combination of laser and anti-VEGF was applied (Table 1). Interestingly, 22.2% (18/81) of eyes were already receiving treatment for ocular hypertension (11.1% were receiving one medication and 4.9% were receiving two medications) (Table 1). In all, 13 eyes had pre-diagnosed glaucoma (n = 6 secondary to steroid response; n = 7 no further definition), 4 eyes had pre-diagnosed open-angle glaucoma and 1 eye was diagnosed with neovascular glaucoma.

**Table 1.** Baseline demographics for diabetic macular edema (DME) patients (81 eyes from 63 patients) with follow-up data.

Parameter	Retro-IDEAL evaluation N = 81
Mean age (range), years	68.0 (36 – 89)
Mean duration of follow-up (± standard deviation (SD)), months	30.8 (± 11.0)
Mean duration since diabetes diagnosis(± SD), years (n = 58)	20.2 (± 14.07)
Type-I and type-II diabetes, n (%)	Type-I, 22 (27.2%) Type-II, 57 (70.4%) Missing information, 2 (2.5%)
Mean duration since DME diagnosis (± SD), years (n = 79)	3.8 (± 2.9)
Incidence of diabetic retinopathy type, % (n = 69)	
Mild non-proliferative	31.8
Moderate non-proliferative	34.8
Severe non-proliferative	17.4
Proliferative	15.9
Pseudophakic eyes	75.3%
Prior vitrectomy, %	48.2
Proportion of eyes that had received treatment for DME in the 12 months pre-FAc implantation, %	
* *Laser	
Any	92.5
Focal	31.5
Panretinal	43.8
Focal and panretinal	24.7
Any intravitreal injection in the 12 months pre-FAc implantation, % (mean injections)	97.5
Anti-VEGF	
Ranibizumab	91.1 (4.3 injections)
Bevacizumab	44.3 (2.8 injections)
Aflibercept	6.3 (5.0 injections)
Corticosteroid	
Triamcinolone	41.8 (1.1 injections)
Dexamethasone	24.1 (1.5 injections)
NSAID	
Ketorolac	2.5
Concomitant treatment of ocular hypertension, %	22.2
1 medication	11.1
2 medications	4.9
Concomitant disease primary system organ class, %	76.5
Vascular disorders	67.9
Cardiac disorders	17.3
Renal and urinary disorders	13.6
Metabolism and nutrition disorders	11.1
(Nervous system disorders)	8.6
Reason for FAc implantation, %	
Insufficient efficacy (i.e. suboptimal) during the pre-treatment phase	100
Strong edema (i.e. persistent)	74.7
No reaction (i.e. response) of visual acuity to the pre-treatment	32.1
High number (i.e. ⩾1 recurrence to first-line therapies) of recurrences during the pre-treatment phase	30.9

### Supplemental therapies to treat DME

During the period of follow-up, 25/81 (30.9%) of eyes received supplemental anti-VEGF or corticosteroid treatment with the majority receiving only an anti-VEGF injection (16/25), 5/25 receiving only a steroid injection and 4/25 receiving a combination of an anti-VEGF and a steroid. Of the 20 eyes (25%) that received an anti-VEGF injection (mean of 4.0 injections (range, 1–12)), aflibercept was most commonly administered (a total of 49 injections in 13 eyes), followed by bevacizumab (a total of 18 injections in 8 eyes) and then ranibizumab (a total of 13 injections in 7 eyes). Six eyes (7.4%) received a steroid and in every case a dexamethasone implant was given (mean of 1 injection). Three (4%) received an additional FAc implant (mean of 1 injection) within 900 days of the first FAc implant; and 4 (5%) received a retreatment with FAc implant treatment (more than 900 days after the first FAc implant). In all, 14/81 eyes (17.3%) received laser treatment (focal or panretinal photocoagulation) with a mean of 1.3 treatments.

### Effectiveness in DME in the full group and those requiring supplemental therapies to treat DME

**Figure 1.**
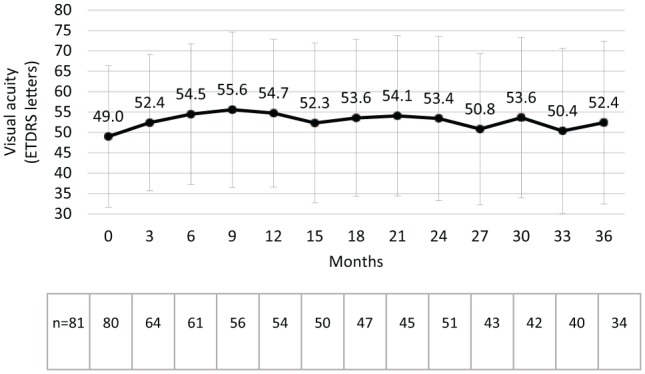
Visual acuity (mean ± SD) following the intravitreal injection of an FAc implant.

**Figure 2.**
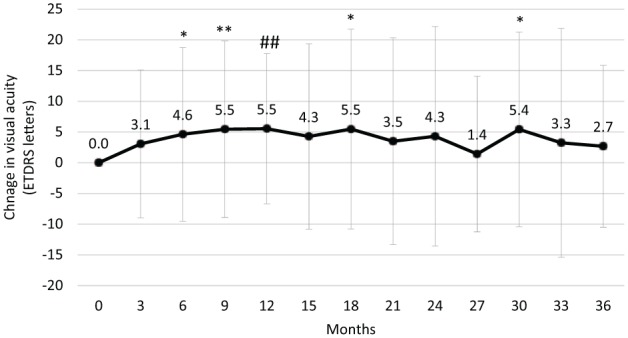
Change in visual acuity (mean ± SD) following the intravitreal injection of an FAc implant. *p ⩽ 0.05, #p ⩽ 0.01, **p ⩽ 0.005, ##p ⩽ 0.001, ***p ⩽ 0.0005, ###p ⩽ 0.0001.

The mean follow-up period was 30.8 ± 11.0 months (88.9% had a follow-up of ⩾30 months). Best-recorded visual acuity (BRVA) was 49.0 ETDRS letters at baseline and numerically improved between months 3 and 36 (Figure 1). Mean changes from baseline are depicted in Figure 2 where the peak change in BRVA was +5.5 letters and occurred at month 9 (P ⩽ 0.005) and maintained at months 18 (P ⩽ 0.05) and 30 (P ⩽ 0.05). Visual outcomes were numerically similar in patients with pseudophakic eyes where a peak increase was seen at month 6 (increasing to 54.0 letters from a baseline of 48.5 letters), which was maintained at 12 (53.0 letters) and 21 months (53.6 letters) before declining slightly at month 36 (50.7 letters). Supplemental therapies were administered in 25 patients with a marked increase occurring in the 2 year – increasing from 4 patients requiring supplemental treatments at month 12 to 10 patients at month 24. In this group, BRVA increased by +5 letters at month 12, and there was an overall loss of BRVA by month 36. In contrast, those that did not require supplemental therapies (n = 57) gained +5.8 letters at 12 months, after which time there was a peak at month 18 (+9.0 letters) before settling back to +5.4 letters at month 36.

**Figure 3.**
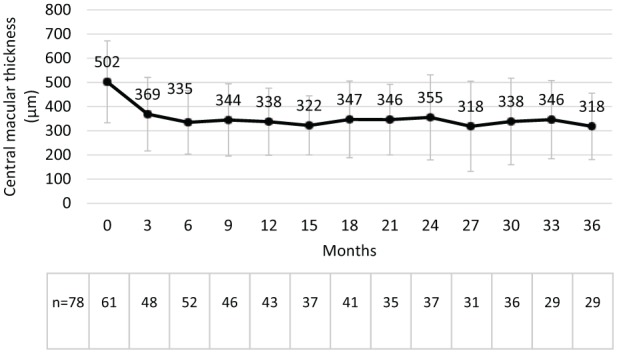
Macular thickness (mean ± SD) following the intravitreal injection of an FAc implant.

**Figure 4.**
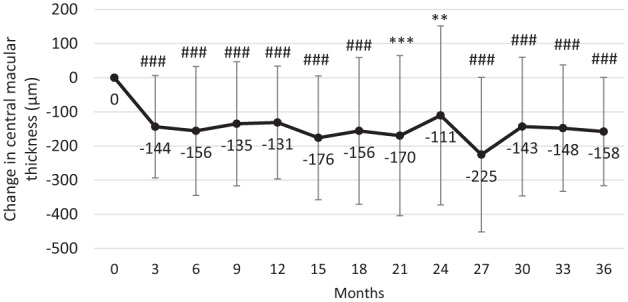
Change in macular thickness (mean ± SD) following the intravitreal injection of an FAc implant. *p ⩽ 0.05, #p ⩽ 0.01, **p ⩽ 0.005, ##p ⩽ 0.001, ***p ⩽ 0.0005, ###p ⩽ 0.0001.

There were also concurrent improvements in CMT with a reduction of CMT from 502 µm at baseline to 338 µm at 12 months, 355 µm at 24 months and 318 µm at 36 months (Figure 3). Significant improvements in CMT were observed at every point from month 3 (P < 0.05 for each point). The mean change from baseline in CMT was −131 µm at 12 months (P ⩽ 0.0001), −111 µm at 24 months (P ⩽ 0.005, n = 30) and −158 µm at 36 months (P ⩽ 0.0001; Figure 4). The effect of supplemental therapies was not so apparent with marked improvements observed at month 3 (−132.0 and −151.7 µm (with and without supplemental therapies)), which were sustained through to month 36 (−134.2 and −173.3 µm, respectively).

### Safety and tolerability in all DME patients

**Figure 5.**
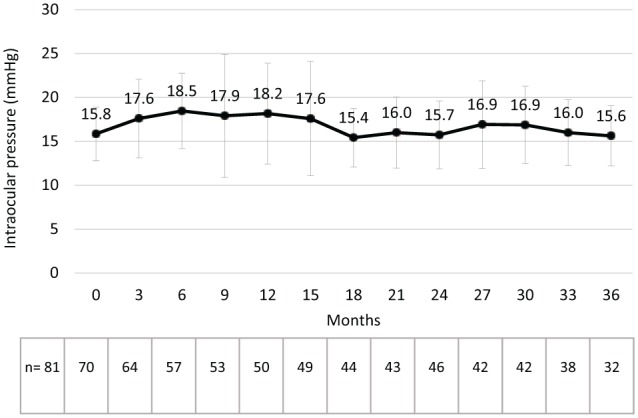
Intraocular pressure (mean ± SD) following the intravitreal injection of an FAc implant.

**Figure 6.**
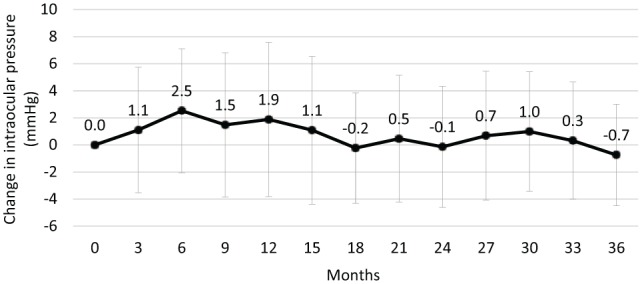
Change in intraocular pressure (mean ± SD) following the intravitreal injection of an FAc implant.

Mean IOP was 15.8 mm Hg at baseline, 18.2 mm Hg at 12 months, 15.7 mm Hg at 24 months and 15.6 mm Hg at 36 months (Figure 5). The mean change from baseline in IOP was +1.9 mm Hg at 12 months, −0.1 mm Hg at 24 months and −0.7 mm Hg at 36 months (Figure 6). In total, 12.3% of patients had an IOP ⩾30 mm Hg at some point during the follow-up period and 22.2% had an increase in IOP of ⩾10 mm Hg (Table 2). The proportion of patients receiving treatment to lower their IOP increased from 22.2% at baseline (Table 1) and 27.2% received IOP-lowering treatment after the FAc implant had been administered (Table 2; treatments included acetazolamide, apraclonidine, bimatoprost, brimonidine, brinzolamide, clonidine, dorzolamide, latanoprost, mannitol, pilocarpine, tafluprost, timolol and travoprost). Three (3.7%) patients had surgery to manage elevated IOP and there was an established response to one patient as they had experienced ocular hypertension prior to having been treated with the FAc implant. Interestingly, there were five eyes that required IOP-lowering drops prior to ILUVIEN but then required no further treatment once the FAc implant had been administered.

**Table 2.** Intraocular pressure outcomes.

Parameter	Patients (%)
⩾30 mm Hg	12.3
>30 mm Hg	7.4
Increase of ⩾10 mm Hg	22.2
IOP treatment before FAc implant	22.2
IOP treatment after FAc implant	27.2
Overal IOP treatments	49.4
Intraocular pressure surgery	3.7%

Four cases (from two patients) of open-angle glaucoma were reported at baseline, even though this is contraindicated in the licence for FAc implant. The cases were continuously treated with IOP medication before treatment with FAc implant. The IOP level was controlled by medication throughout the therapy, and VA and CMT were improved or stabilised after the FAc implant. IOP-lowering surgery was performed in three of the four cases once vision has started to depreciate (n = 2) or CMT has started to worsen (n = 1). All eyes improved after surgery and no surgeries were related to IOP increases.

Overall, 21.3% (17/80 (1 missing)) of phakic eyes were reported to have a cataract, whereas 59% (n = 10/17) had cataract already at baseline. Phakic patients without cataract diagnosis at baseline (n = 7) developed a cataract at a mean of 254 days and in 5 the cataract was removed. Phakic patients with cataract at baseline underwent cataract surgery at a mean of 403 days.

### Other adverse events in DME patients

Known drug-related adverse events are reported in the safety paragraph above. In addition to these, there was one DME patient (aged 69 years of age with type-1 diabetes, proliferative DR, an epiretinal membrane and a CMT of 900 µm at baseline) where the FAc implant was removed by pars plana vitrectomy, after 396 days, after IOP reached a value of 38 mm Hg. This was despite being treated with IOP-lowering medication and the patient’s VA and CMT improving after the FAc implant was administered. There were also no reported cases of endophthalmitis.

### Case study illustrating the use of the fluocinolone acetonide implant

**Figure 7.**
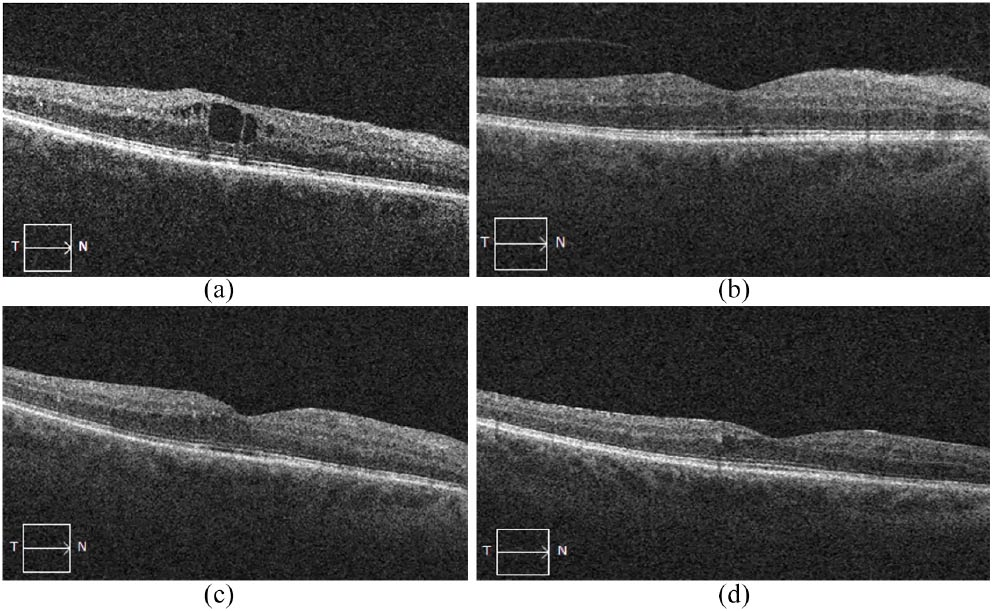
Case study: OCT images of the right eye on the day the fluocinolone acetonide implant was administered (CMT, 406 µm; BCVA, 34 letters) (a) and then at 13 weeks post-FAc implant: CMT, 332 µm; BCVA, 83 letters (b), 64 weeks post-FAc implant: CMT, 319 µm; BCVA, 88 letters (c) and 163 weeks post-FAc implant: CMT 272 µm; BCVA, 83 letters (d).

A 51-year-old man with type-II diabetes mellitus developed DME in both his eyes in 2011. The patient received prior treatment, which included laser (panretinal laser photocoagulation), anti-VEGF (3 injections of ranibizumab and 2 injections of bevacizumab) and steroid (1 injection of triamcinolone and dexamethasone). Following a suboptimal response to these prior DME treatments, the patient was treated with the FAc implant on 14 May 2013. On the day of implantation, VA was 34 letters and CMT was 406 µm, and both improved to 83 letters and 272 µm on 29 June 2016 (see Figure 7).

## Discussion

To the authors’ knowledge, real-world data after treatment with an FAc implant have so far been limited to reports with limited follow-up.^15–21^ This is the first known report detailing results over a 3-year follow-up period (a mean of 30.8 months) and confirms that treatment with an FAc implant offers clinically significant improvements in VA and macular thickness over the lifespan of the implant. Most of the benefits in VA and macular thickness occurred by the 3- and 6-month evaluations, and these benefits were sustained up to year 3.

The current study outlines a number of significant differences between the patients in this study and those in the FAME trials, therefore highlighting the difference between patients treated in clinical practices as opposed to those selected for randomised controlled trials. In the FAME trials, patients were insufficiently responsive to prior laser, whereas current patients were insufficiently responsive to primarily intravitreal therapies (i.e. intravitreal injections of anti-VEGF and corticosteroids) prior to receiving ILUVIEN. Also, patients in the real world had more ‘severe DME’ owing to the baseline characteristics and prior treatment history.

It is also noteworthy that about half of the patients in real-world practice had been treated with prior focal laser, which was then combined with other treatments. This may indicate that focal laser alone was ineffective in the treatment of DME in this population. It was also apparent that in real-world practice, baseline VA was more variable (range, 20–80 letters) as compared with the FAME trials where patients were only included if they had a baseline VA between ⩾19 and ⩽68 letters in the study eye. The inclusion of patients based on baseline VA is also reported in other randomised controlled trials with other DME agents. The mean age in real-world practice was also higher than in the FAME trials with a significant proportion (8 out of 10) being older than 70 years. In all, 8 out of the 10 patients in this real-world practice study had a BMI in excess of 25 kg/m^2^ and had a relatively high cardiovascular risk profile. The proportion of patients (1 in 3) also had severe non-proliferative or proliferative DR at baseline and they had a mean DME duration in excess of 4 years.

Thus, there are notable differences between the patients in this study and those in the FAME trials, which limit meaningful comparisons of the efficacy in these two evaluations. In the case of tolerability, however, such comparisons are potentially more meaningful as the therapy was newly approved in the last 5 years. The IOP outcomes were similar to those in the FAME trials. Further analysis of these data also demonstrated that mean changes in IOP were greatest within the first 6 months after implantation (a peak change of +2.5 mm Hg at month 6). These changes gradually subsided thereafter, returning to baseline levels by month 18 and remaining no more than 1 mm Hg above baseline levels until the end of the follow-up period at year 3. More than 50% of the cases of elevated IOP were managed topically (49.4% in total, of which 27.2% were emergent IOP cases) and only 3 cases required surgery. In the FAME trials, patients were excluded if they had glaucoma, ocular hypertension, IOP >21 mm Hg or concurrent therapy with IOP-lowering agents in the study eye.^22^ In real-world practice, however, a small number of patients were included with pre-existing glaucoma, even though this is contraindicated in the approved summary of product characteristics in Europe^10^ and a significant proportion of patients were receiving IOP-lowering drops (22.2%) before ILUVIEN was administered. In light of this, the present study results support the long-term tolerability of the FAc implant in everyday clinical practice as it was in the clinical trials.

Effectiveness results were also very promising, considering that no patients were excluded and no algorithms were used to impute data. This means that all patients selected for treatment were included irrespective of the physicians’ difficulty in treating the disease. By year 3, mean VA was shown to increase from 49.0 to 52.4 letters with a peak effect reported at month 9 (55.6 letters) and a sustained effect (~54.0 letters) being observed through to month 30 (Figure 1). This was accompanied by a reduction in CMT, which started at 502.0 µm and remained ⩽ 369.0 µm between months 3 and 36 (see Figure 3).

There are only a limited number of studies reporting 3-year outcomes following ILUVIEN treatment. Bailey et al.^15^ reported the results of a UK audit of electronic medical records from 14 centres where ILUVIEN has been used in the National Health Service. This study was published in 2017 and is currently the largest reported real-world dataset to date. It includes 305 patients (345 DME eyes) with up to 2 years follow-up. The authors reported improvements in both mean VA (from 51.9 to 57.2 letters) and central subfield foveal thickness (reduction from 451.2 to 355.5 μm). Fusi-Rubiano et al.^23^ reported outcomes at 3 years and also showed concurrent improvements in VA and central retinal thickness. Between baseline and year 2, VA improved from 46.5 to 53.0 letters and central retinal thickness improved from 451 to 342 μm. These effects were sustained through to year 3 (to 57.2 letters and 314 μm, respectively). It is important to mention that 22 eyes were followed up to year 2 but only 6 of these had completed 3 years at the time of the report. This means that outcomes in the larger population are still pending and are needed to enable a fair comparison with the current data set and to also assess the effect of supplemental therapies on reported outcomes. Current results highlight the sustained effects being achieved with the FAc implant in real-world practices in Germany and that these are similar in magnitude and nature to those reported by Bailey et al.^15^ and Fusi-Rubiano et al.^23^ Baseline values are below (i.e. 502 µm and 49 letters) those reported in the UK studies. This further highlights the longer duration of DME of the current cohort of patients.

It is important to appreciate that the patients in this analysis were some of the very first patients treated in Germany following the approval of ILUVIEN. They had a pronounced inflammatory disease state as indicated by both the long duration of DME and the heavy course of pre-treatments (for DME and IOP) before receiving the FAc implant. It is also apparent that the FAc implant was administered as a second-line therapy after patients had an insufficient response to the current standard of care (largely anti-VEGF therapy administered with laser or intravitreal steroid). This is explained by the fact that when the FAc implant was commercially available in European markets in 2013, there was a lack of other appropriate therapeutic options available. Therefore, patients were more likely to have already received a higher number of prior treatments for prolonged period. It is also likely that these treatments were continued in the absence of a sufficient response when the underlying disease was likely to have progressed to a more pro-inflammatory state, as reflected by the starting CMT (>500 µm at baseline). Furthermore, there is a question as to whether the more optimal outcomes could be achieved in these patients. Indeed, the EARLY study^7^ showed that within 3 months of starting anti-VEGF therapy, it is possible to evaluate whether or not a patient is responding adequately to anti-VEGF treatment (specifically ranibizumab) and this is predictive of how a patient will respond over a 3-year period. Therefore, physicians have an opportunity at this early stage to consider switching insufficiently responding patients from anti-VEGF to a corticosteroid treatment and potentially achieve better outcomes for their patients by (1) avoiding unnecessary damage to the retina and (2) providing a steroid that targets both anti-VEGF and anti-inflammatory mechanisms underlying DME progression.

Although the rigorously controlled conditions mandated in clinical trial protocols facilitate well-controlled evaluations of potential effectiveness, a very different environment exists in everyday clinical practice. A broader cross-section of patients will be encountered in everyday clinical practice with a greater range of comorbidities – and more variation in baseline values for VA, macular thickness and IOP – than may be allowed in a clinical trial protocol. Another variable in the current study is the physicians’ reasons for using ILUVIEN to treat DME. In all cases, the physician had treated the patient because of an insufficient efficacy with previous treatment which was primarily due to ‘treatment of persistent edema’ but also included ‘no change in VA after previous treatment’ and a ‘high number of recurrences after previous treatment’. Another variable is that patients may not have been monitored so closely (i.e. every quarter as defined in the product’s summary of product characteristics^10^) and injection schedules may not be adhered to so closely (i.e. due to the underlying need for patients to attend many ongoing injections and appointments to manage their eye condition).^24^ It is also important to note that, at the time of the pivotal study for the FAc implant (the FAME study),^11^ anti-VEGF therapy was not yet available and thus no patients had already received such a therapy. In contrast, in the current standard of clinical care, it is well-documented that the majority of patients with DME had been exposed to first-line anti-VEGF therapy^15,25,26^ – an observation that is also confirmed in the current study.

## Conclusion

This report provides the longest duration of follow-up with the FAc implant in everyday clinical practice in Germany and confirms that the long duration of action reported in the pivotal FAME study^11^ is also achieved in real world. Patients who had previously experienced suboptimal outcomes with anti-VEGF and other DME therapies achieved sustained improvements in VA and macular thickness lasting for up to 36 months after receiving a single FAc implant. Furthermore, the incidence of treatment-emergent IOP elevation tended to be similar to that reported in the FAME study, thus highlighting the good benefit-to-risk profile even after an insufficient response to repeated prior injections of primarily anti-VEGF. These clinically significant outcomes were achieved even in this relatively difficult-to-treat and high-risk patient population and support the FAME studies by demonstrating that steroids, specifically FAc in this study, are an effective and tolerable treatment option in patients with DME. This study also highlights that the FAc implant has largely been used after multiple prior therapies and not selected after the first insufficient response to an anti-VEGF. This study highlights the need for better patient selection and potentially how early detection of insufficient responses to intravitreal anti-VEGF may be used in the clinic to optimise outcomes in patients with persistent or recurrent DME.
